# Simultaneous Identification of Multiple Driver Pathways in Cancer

**DOI:** 10.1371/journal.pcbi.1003054

**Published:** 2013-05-23

**Authors:** Mark D. M. Leiserson, Dima Blokh, Roded Sharan, Benjamin J. Raphael

**Affiliations:** 1 Department of Computer Science and Center for Computational Molecular Biology, Brown University, Providence, Rhode Island, United States of America; 2 Blavatnik School of Computer Science, Tel-Aviv University, Tel-Aviv, Israel; ETH Zurich, Switzerland

## Abstract

Distinguishing the somatic mutations responsible for cancer (*driver* mutations) from random, *passenger* mutations is a key challenge in cancer genomics. Driver mutations generally target cellular signaling and regulatory pathways consisting of multiple genes. This heterogeneity complicates the identification of driver mutations by their recurrence across samples, as different combinations of mutations in driver pathways are observed in different samples. We introduce the Multi-Dendrix algorithm for the simultaneous identification of multiple driver pathways *de novo* in somatic mutation data from a cohort of cancer samples. The algorithm relies on two combinatorial properties of mutations in a driver pathway: high coverage and mutual exclusivity. We derive an integer linear program that finds set of mutations exhibiting these properties. We apply Multi-Dendrix to somatic mutations from glioblastoma, breast cancer, and lung cancer samples. Multi-Dendrix identifies sets of mutations in genes that overlap with known pathways – including Rb, p53, PI(3)K, and cell cycle pathways – and also novel sets of mutually exclusive mutations, including mutations in several transcription factors or other genes involved in transcriptional regulation. These sets are discovered directly from mutation data with *no prior knowledge* of pathways or gene interactions. We show that Multi-Dendrix outperforms other algorithms for identifying combinations of mutations and is also orders of magnitude faster on genome-scale data. Software available at: http://compbio.cs.brown.edu/software.

## Author Summary

Cancer is a disease driven largely by the accumulation of somatic mutations during the lifetime of an individual. The declining costs of genome sequencing now permit the measurement of somatic mutations in hundreds of cancer genomes. A key challenge is to distinguish *driver mutations* responsible for cancer from random *passenger* mutations. This challenge is compounded by the observation that different combinations of driver mutations are observed in different patients with the same cancer type. One reason for this heterogeneity is that driver mutations target signaling and regulatory pathways which have multiple points of failure. We introduce an algorithm, Multi-Dendrix, to find these pathways solely from patterns of mutual exclusivity between mutations across a cohort of patients. Unlike earlier approaches, we simultaneously find multiple pathways, an essential feature for analyzing cancer genomes where multiple pathways are typically perturbed. We apply our algorithm to mutation data from hundreds of glioblastoma, breast cancer, and lung adenocarcinoma patients. We identify sets of interacting genes that overlap known pathways, and gene sets containing subtype-specific mutations. These results show that multiple cancer pathways can be identified directly from patterns in mutation data, and provide an approach to analyze the ever-growing cancer mutation datasets.

## Introduction

Cancer is a disease driven in part by somatic mutations that accumulate during the lifetime of an individual. The declining costs of genome sequencing now permit the measurement of these somatic mutations in large numbers of cancer genomes. Projects such as The Cancer Genome Atlas (TCGA) and International Cancer Genome Consortium (ICGC) are now undertaking this task in hundreds of samples from dozens of cancer types. A key challenge in interpreting these data is to distinguish the functional *driver* mutations important for cancer development from random *passenger* mutations that have no consequence for cancer. The ultimate determinant of whether a mutation is a driver or a passenger is to test its biological function. However, because the ability to detect somatic mutations currently far exceeds the ability to validate experimentally their function, computational approaches that predict driver mutations are an urgent priority. One approach is to directly predict the functional impact of somatic mutations using additional biological knowledge from evolutionary conservation, protein structure, etc. and a number of methods implementing this approach have been introduced (see [1]–[4]). These methods are successful in predicting the impact of some mutations, but generally do not integrate information across different types of mutations (single nucleotide, indels, larger copy number aberrations, etc.); moreover, these methods are less successful for less conserved/studied proteins.

Given the declining costs of DNA sequencing, a standard approach to distinguish driver from passenger mutations is to identify *recurrent* mutations, whose observed frequency in a large cohort of cancer patients is much higher than expected [5], [6]. Nearly all cancer genome sequencing papers, including those from TCGA [7]–[10] and other projects [5], [11], [12], report a list of significantly mutated genes. However, driver mutations vary greatly between cancer patients – even those with the same (sub)type of cancer – and this heterogeneity significantly reduces the statistical power to detect driver mutations by tests of recurrence. One of the main biological explanations for this mutational heterogeneity is that driver mutations target not only individual genomic loci (e.g. nucleotides or genes), but also target groups of genes in cellular signaling and regulatory pathways. Consequently, different cancer patients may harbor mutations in different members of a pathway important for cancer development. Thus, in addition to testing individual loci, or genes, for recurrent mutation in a cohort of patients, researchers also test whether groups of genes are recurrently mutated. Since exhaustive testing of all groups of genes is not possible without prohibitively large sample sizes (due to the necessary multiple hypothesis testing correction), current approaches focus on groups of genes defined by prior biological knowledge, such as known pathways (e.g. from KEGG [13]) or functional groups (e.g. from GO [14]), and methods have been introduced to look for enrichment in such pre-defined groups of genes (e.g. [15]–[17]). More recently, methods that identify recurrently mutated subnetworks in protein-protein interaction networks have also been developed, such as NetBox [18], MeMO [19], HotNet [20], and EnrichNet [21].

Knowledge of gene and protein interactions in humans remain incomplete, and most existing pathway databases and interaction networks do not precisely represent the pathways and interactions that occur in a particular cancer cell. Thus, restricting attention to only those combinations of mutations recorded in these data sources may limit the possibility for novel biological discoveries. Thus algorithms that do not make this restriction – but also avoid the multiple hypothesis testing problems associated with exhaustive enumeration – are desirable. Recently, the RME [22] and De novo Driver Exclusivity (Dendrix) [23] algorithms were introduced to discover *driver pathways* using combinatorial constraints derived from biological knowledge of how driver mutations appear in pathways [24], [25]. In particular, each cancer patient contains a relatively small number of driver mutations, and these mutations perturb multiple cellular pathways. Thus, each driver pathway will contain approximately one driver mutation per patient. This leads to a pattern of *mutual exclusivity* between mutations in different genes in the pathway. In addition, an important driver pathway should be mutated in many patients, or have high *coverage* by mutations. Thus, driver pathways correspond to sets of genes that are mutated in many patients, but whose mutations are mutually exclusive, or approximately so. We emphasize that the *driver pathways exhibiting patterns of mutually exclusivity and high coverage are generally smaller and more focused* than most pathways annotated in the literature and pathway databases. The latter typically contain many genes and perform multiple different functions; e.g. the “cell cycle” pathway in KEGG contains 143 genes. It is well known that co-occurring (i.e., not exclusive) mutations are observed in these larger, multifunctional biological pathways [25]. The RME and Dendrix algorithms use different approaches to find sets of genes with high coverage and mutual exclusivity: RME builds sets of genes from pairwise scores of exclusivity, while Dendrix computes a single score for the mutual exclusivity of a set of genes, and finds the highest scoring set. The aforementioned MeMO algorithm [19] also considers mutual exclusivity between mutations, but only for pairs of genes that have recorded interactions in a protein-protein interaction network. Thus, MeMO does not attempt to identify driver pathways *de novo* and can only define subnetworks in existing interaction networks. While many of the strongest signals of mutual exclusivity are between genes with known interactions, below we show examples in cancer data of mutual exclusive mutations between genes with no known direct iterations.

The two existing *de novo* algorithms, RME and Dendrix, consider the detection of only a *single* driver pathway from the pattern of mutual exclusivity between mutations. However, it is well known that mutations in several pathways are generally required for cancer [26]. There is little reason to assume that mutations in different pathways will be mutually exclusive, and in contrast may exhibit significant patterns of co-occurrence across patients. Multiple pathways may be discovered using these algorithms by running the algorithm iteratively, removing the genes found in each previous iteration, and such an approach was employed for Dendrix [23]. However, such an iterative approach is not guaranteed to yield the optimal set of pathways.

Here we extend the Dendrix algorithm in three ways. First, we formulate the problem of finding exclusive, or approximately exclusive, sets of genes with high coverage as an integer linear program (ILP). This formulation allows us to find optimal driver pathways of various sizes directly – in contrast to the greedy approximation and Markov Chain Monte Carlo algorithms employed in Dendrix. Second, we generalize the ILP to *simultaneously* find *multiple* driver pathways. Third, we augment the core algorithm with additional analyses including: examining gene sets for subtype-specific mutations, summarizing stability of results across different number and size of pathways, and imposing greater exclusivity of gene sets.

We apply the new algorithm, called Multi-Dendrix, to four somatic mutation datasets: whole-exome and copy number array data in 261 glioblastoma (GBM) patients from The Cancer Genome Atlas (TCGA) [7], whole-exome and copy number array data in 507 breast cancer (BRCA) patients from TCGA [8], 601 sequenced genes in 84 patients with glioblastoma multiforme (GBM) from TCGA [7] and 623 sequenced genes in 188 patients with lung Adenocarcinoma [27]. In each dataset Multi-Dendrix finds biologically interesting groups of genes that are highly exclusive, and where each group is mutated in many patients. In all datasets these include groups of genes that are members of known pathways critical to cancer development including: Rb, p53, and RTK/RAS/PI(3)K signaling pathways in GBM and p53 and PI(3)K/AKT signaling in breast cancer. Multi-Dendrix successfully recovers these pathways solely from the pattern of mutual exclusivity and *without any prior information* about the interactions between these genes. Moreover, Multi-Dendrix also identifies mutations that are mutually exclusive with these well-known pathways, and potentially represent novel interactions or crosstalk between pathways. Notable examples include mutual exclusivity between: mutations in PI(3)K signaling pathway and amplification of PRDM2 (and PDPN) in glioblastoma; mutations in p53, GATA3 and cadherin genes in breast cancer.

Finally, we compare Multi-Dendrix to an alternative approach of iteratively applying Dendrix [23] or RME [22], two other algorithms that search for mutually exclusive sets. We show that these iterative approaches typically fail to find an optimal set of pathways on simulated data, while Multi-Dendrix finds the correct pathways even in the presence of a large number of false positive mutations. On real cancer sequencing data, the groups of genes found by Multi-Dendrix include more genes with known biological interactions. Moreover, Multi-Dendrix is orders of magnitude faster than these other algorithms, allowing Multi-Dendrix to scale to the latest whole-exome datasets on hundreds of samples, which are largely beyond the capabilities of Dendrix and RME. Multi-Dendrix is a novel and practical approach to finding multiple groups of mutually exclusive mutations, and complements other approaches that predict combinations of driver mutations using biological knowledge of pathways, interaction networks, protein structure, or protein sequence conservation.

## Results

### Multi-Dendrix algorithm

The Multi-Dendrix algorithm takes somatic mutation data from 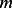 cancer patients as input, and identifies *multiple* sets of mutations, where each set satisfies two properties: (1) the set has high *coverage* with many patients having a mutation in the set; (2) the set exhibits a pattern of mutual exclusivity where most patients have exactly one mutation in the set. We briefly describe the Multi-Dendrix algorithm here. Further details are provided in the Methods section below.

We assume that somatic mutations have been measured in 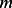 cancer patients and that these mutations are divided into 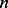 different *mutation classes*. A mutation class is a grouping of different mutation types at a specific genomic locus. In the simplest case, a mutation class corresponds to a grouping of all types of mutations (single nucleotide variants, copy number aberrations, etc.) in a single gene. We represent the somatic mutation data as an 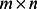 binary *mutation matrix* 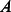, where the entry 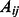 is defined as follows:
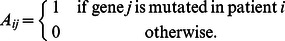
(1)


More generally, a mutation class may be defined for an arbitrary genomic locus, and not just a gene, and may distinguish different types of mutations. For example, one may define a mutation class as single-nucleotide mutations in an individual residue in a protein sequence or in a protein domain. Or alternatively, one may separate different types of mutations in a gene (e.g. single-nucleotide mutations, deletions, or amplifications) by creating separate mutation classes for each mutation type in each gene. We will use this later definition of mutation classes in the results below. For ease of exposition we will assume for the remainder of this section that each mutation class is a gene.

Vandin et al. [23] formulate the problem of finding a set of genes with high coverage and high exclusivity as the *Maximum Weight Submatrix Problem*. Here the weight 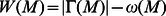 of a set 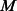 of genes is the difference between the coverage 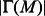, the number of patients with a mutation in one of the genes in 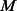, and the coverage overlap 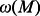, the number of patients having a mutation in more than one gene in 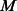. Vandin et al. [23] introduce the De novo Driver Exclusivity (Dendrix) algorithm [23] that finds a set 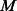 of 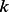 genes with maximum weight 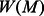.

While finding single driver pathways is important, most cancer patients are expected to have driver mutations in multiple pathways. Dendrix used a greedy iterative approach to find multiple gene sets (described below), that is not guaranteed to find optimal gene sets. Identification of multiple driver pathways requires a criterion to evaluate possible collections of gene sets. Appealing to the same biological motivation as above, we expect that each pathway contains approximately one driver mutation. Moreover, since each driver pathway is important for cancer development, we also expect that most individuals contain a driver mutation in most driver pathways. Thus, we expect high exclusivity within the genes of each pathway and high coverage of each pathway on its own. One measure that satisfies these criteria is to find a *collection* 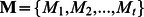 of gene sets whose sum of weights is maximized.

We define the Multiple Maximum Weight Submatrices problem as the problem of finding such a maximum weight collection. We solve the *Multiple Maximum Weight Submatrix* problem using an integer linear program (ILP), and refer to the resulting algorithm as Multi-Dendrix (see Methods). In addition, the ILP formulation used in Multi-Dendrix uses a modified weight function 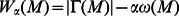, where 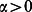 is a parameter that adjusts the tradeoff between finding sets with higher coverage 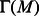 (more patients with a mutation) versus higher coverage overlap 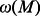 (greater non-exclusivity between mutations). We use this parameter in the breast cancer dataset below. In contrast, Dendrix was limited to 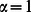.

### Simulated data

We compare Multi-Dendrix to iterative versions of Dendrix [23] and RME [22] on simulated mutation data with both driver mutations implanted in pathways in a mutually exclusive manner and random passenger mutations. The goal of these simulations is to compare Multi-Dendrix to other algorithms that identify mutually exclusive genes on straightforward datasets that contain *multiple* mutually exclusive sets. We generate mutation data for 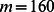 patients and 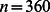 genes as follows. We select a set of four pathways 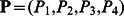 with each 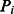 containing four genes. We select the coverage 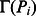 uniformly from the following intervals: 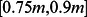, 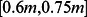, 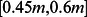, 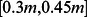, respectively. The size of this dataset and the varying coverages of the pathways model what is observed in real data (see § Somatic Mutation data) and is consistent with models of mutation progression where driver mutations accumulate in pathways [28]. For each pathway 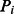, we select 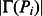 patients at random and add a driver mutation to exactly one gene from the set 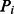. Thus, the driver mutations in each pathway are mutually exclusive. We then add passenger mutations by randomly mutating genes in each patient with probability, 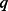, the *passenger mutation probability*.We used values of 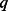 similar to our estimates for 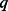 on the TCGA GBM and Lung cancer data sets (in § Somatic Mutation data below), which were 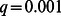 and 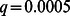, respectively. We emphasize that these simulations do not model all of the complexities of somatic mutations in cancer e.g. gene-specific and patient-specific mutation rates, genes present in multiple pathways, etc.

Since the Dendrix and RME algorithms are designed to find single pathways, we compared Multi-Dendrix to iterative versions of these methods that return multiple gene sets. For Dendrix we used the iterative approach described in [23]: apply Dendrix to find a highest scoring gene set, remove those genes from the dataset, and apply Dendrix to the reduced dataset, repeating these steps until a desired number 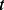 of gene sets are found. We will refer to this algorithm as Iter-Dendrix. Thus, Iter-Dendrix returns a collection 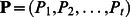 of 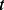 gene sets such that 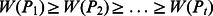. We implemented the analogous iterative version of RME, and will refer to this algorithm as Iter-RME. We compared the collection 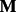 of gene sets found by each algorithm to the planted pathways 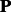, computing the symmetric difference 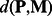 between 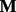 and 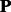 as described in Methods.

Table 1 shows a comparison of Multi-Dendrix, Iter-Dendrix, and Iter-RME on simulated mutation data for different values of 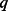. Note that we do not show comparisons to Iter-RME for 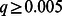 as Iter-RME did not complete after 24 hours of runtime for *any* of the 1000 simulated mutation data sets. While the RME publication [22] analyzed mutation matrices with thousands of genes and hundreds of patients, this analysis (and the released RME software) required that mutations were presented in at least 10% of the samples, greatly reducing the number of genes/samples input to the algorithm. In fact, a threshold of 10% will remove nearly all genes in current whole-exome studies (see § Comparison of Multi-Dendrix and RME).

**Table 1:** A comparison of the algorithms on simulated mutation data with varying passenger mutation probability 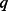.

	Avg. distance 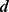 from planted pathways		
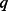	Multi-Dendrix	Iter-RME	Iter-Dendrix
0.0	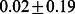	**0.01** 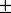 **0.12**	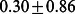
*0.0001*	*0.02* 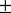 *0.18*	***0.01*** 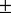 ***0.16***	*0.30* 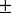 *0.86*
0.0005	**0.04** 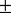 **0.23**	0.10 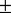 0.40	0.35 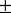 0.89
*0.001*	***0.10*** 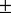 ***0.35***	*0.32* 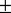 *0.60*	*0.44* 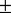 *1.01*
0.005	**0.44** 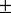 **0.71**	–	0.75 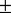 1.07
0.01	**1.03** 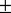 **1.00**	–	1.20 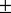 1.15
0.015	**1.68** 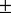 **1.16**	–	1.78 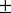 1.26
0.02	**2.17** 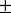 **1.24**	–	2.21 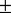 1.29

Italicized rows correspond to values of 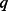 approximated from real cancer datasets. Each entry is mean (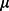) and standard deviation (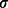) (across 1000 simulations) of the distance 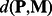 between the planted set of pathways 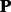 and the collections 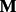 found by each algorithm. The minimum distance 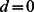 indicates an algorithm found the planted pathways exactly, while the maximum distance 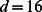 indicates that an algorithm did not find *any* of the genes in the planted pathways. Bold text indicates the top performing algorithm for each value of 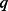. Multi-Dendrix is the top performer for all values of 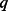 except the smallest 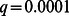. The differences between Multi-Dendrix and both Iter-Dendrix and Iter-RME are statistically significant (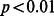) for 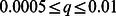. For 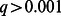, Iter-RME did not complete after 24 hours of runtime.

For 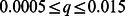, Multi-Dendrix identifies collections of gene sets that were significantly closer (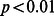) to the planted pathways 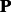 than the collections found by either Iter-Dendrix and Iter-RME. These results demonstrate that Multi-Dendrix outperforms other methods, even when the passenger mutation probability 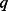 is more than 15 times greater than the value estimated from real somatic mutation data. For 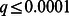, the differences between Multi-Dendrix and Iter-RME were not significant.

We also compared the runtimes of each algorithm on the simulated datasets. Multi-Dendrix was several orders of magnitude faster than Iter-Dendrix and Iter-RME on all datasets (Table 2). Note that as the passenger mutation probability 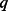 increases, the number of recurrently mutated passenger genes increases. Multi-Dendrix scales much better than Iter-RME and maintains a significant advantage over Iter-Dendrix, completing all simulated datasets in less than 5 seconds.

**Table 2:** A comparison of the runtimes of Multi-Dendrix, Iter-RME, and Iter-Dendrix on simulated mutation data with varying passenger mutation probability 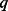.

	Avg. runtime (secs)		
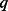	Multi-Dendrix	Iter-RME	Iter-Dendrix
*0.0001*	***0.28***	*19.22*	*609.79*
0.0005	**0.28**	17.36	621.22
*0.001*	***0.50***	*123.16*	*610.21*
0.005	**1.46**	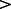 86, 400	672.43
0.01	**2.60**	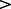 86, 400	711.55
0.015	**4.06**	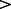 86, 400	730.85
0.02	**4.93**	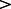 86, 400	727.82

Runtimes for each algorithm are reported as the mean runtime of 10 runs for each value of 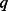. Note that Multi-Dendrix has runtimes under 5 seconds for all datasets, and is orders of magnitude faster than the other methods for each 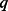. For Iter-RME, we report a runtime of 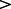
86,400 seconds for 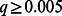 as Iter-RME did not complete within one day. Simulations were performed on machines running 64-bit Debian Linux with Xeon 2.8 GHz processors and a maximum of 8 GB of available memory.

We evaluated how the runtime of Multi-Dendrix scales to larger datasets. Using the same passenger mutation probabilities 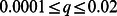 listed above, we calculated the average runtime in seconds of Multi-Dendrix for ten simulated mutation matrices with 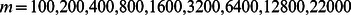 genes and 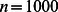 patients, more than the number of patients to be measured in any cancer study from TCGA. In each case, we run Multi-Dendrix only on the subset of genes that are mutated in more than the expected number 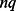 of samples. For the largest dataset with 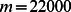 genes, the average number of genes input to Multi-Dendrix for the highest and lowest passenger mutation probabilities are 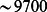 and 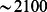, respectively. (Table S1 shows the average number of input genes for varying 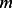 and 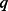.) The average runtime for this largest dataset is under one hour (average of 54.4 minutes). Figure S1 shows the runtimes for varying 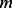 and 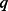.

### The Multi-Dendrix Computational Pipeline

**Figure 1:**
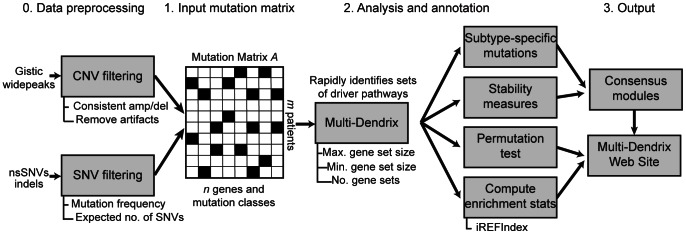
The Multi-Dendrix pipeline. Multi-Dendrix analyzes integrated mutation data from a variety of sources including single-nucleotide mutations and copy number aberrations. Multiple gene set are identified using a combinatorial optimization approaches. The output is analyzed for subtype-specific mutations and summarized across multiple values of the parameters: 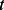, number of gene sets, and 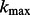, maximum size per gene set.

We incorporate the Multi-Dendrix algorithm into a larger pipeline (Figure 1) that includes several additional pre- and post-processing tasks including: (1) Building mutation matrices for input into Multi-Dendrix; (2) Summarizing Multi-Dendrix results over multiple values for the parameters 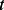, the number of gene sets, 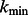 the minimum size of a gene set, and 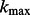 the maximum size of a gene set; (3) Evaluating the statistical significance of results; (4) Examining Multi-Dendrix results for mutually exclusive sets resulting from subtype-specific mutations. We describe these steps briefly below, with further details in the Methods and Supporting Information.

First, we build mutation matrices 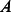 from somatic mutation data. We use several steps to process single-nucleotide variant (SNV) data, copy number variant (CNV) data, and to combine both types of data. Second, in contrast to simulated data, on real data we do not know the correct values of the parameters 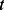, 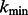, and 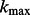. Thus, we consider a reasonable range of values for these parameters and summarize the results over these parameters into *modules*. We build a graph, where the nodes are individual genes (or mutation classes) and edges connect genes (respectively mutation classes) that appear in the same gene set for more than one value of the parameters. We weight each edge with the fraction of parameter values for which the pair of genes appear in the same gene set. The resulting edge-weighted graphs provide a measure of the stability of the resulting gene sets over different parameter values. By choosing a minimum edge weight, we partition the graph into connected components, or *modules*. One may choose to use these modules as the output of Multi-Dendrix.

Third, we evaluate the statistical significance of our results using two measures. Since the collection 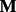 with high weight 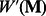 may not be surprising in a large mutation matrix 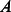, the first measure evaluates the significance of the score 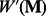 maximized by Multi-Dendrix. We evaluate whether the weight 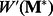 of the maximum weight collection 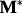 output by Multi-Dendrix is significantly large compared to an empirical distribution of the maximum weight sets from randomly permuted mutation data. We generate random mutation data using the permutation test described in [19]. This test permutes the mutations among the genes in each patient, preserving both the number of mutated genes in each patient and the number of patients with a mutation in each gene while perturbing any patterns of exclusivity between mutated genes. Note that this permutation test requires running Multi-Dendrix many times to determine statistical significance for a single parameter setting. Thus, the runtime advantages of Multi-Dendrix compared to Iter-Dendrix and Iter-RME are very important in practice on real datasets.

Next, we evaluate whether the collection 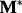 output by Multi-Dendrix contains more protein-protein interactions than expected by chance by applying our *direct interactions test* on a PPI network constructed from the union of the KEGG and iRefIndex PPI networks. The direct interactions test computes a statistic 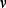 of the difference in the number of interactions *within* and *between* gene sets in 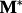, and compares the observed value of 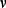 to an empirical distribution on 1000 permuted PPI networks (full details of the test are in 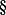 Evaluating known interactions). These permuted networks account for the observation that many genes that are frequently mutated in cancer also have large degree in the interaction network – either due to biological reasons or ascertainment bias. We use an interaction network to assess biological function rather than known pathways (e.g. KEGG pathways or GSEA sets) because most of these pathways are relatively large, while the gene sets found by Multi-Dendrix that exhibit exclusivity tend to be much smaller, each containing only a few genes.

Finally, we examine possible correlations between the mutually exclusive sets reported by Multi-Dendrix and particular subsets of samples. A number of cancers are divided into subtypes according to pathology, cytogenetics, gene expression, or other features. Since mutations that are specific to particular subtypes will be mutually exclusive, disease heterogeneity is an alternative explanation to pathways for observed mutually exclusive sets. For example, [29] report four subtypes of GBM based on gene expression clusters, and show that several mutations – including IDH1, PDGFRA, EGFR, and NF1 – have strong association with individual subtypes. Unfortunately, if the subtypes are unknown there is no information for Multi-Dendrix, Dendrix, RME, or other algorithms that analyze mutual exclusivity to distinguish between mutual exclusivity resulting from subtypes and mutual exclusivity resulting from pathways or other causes. If subtypes are known, two possible solutions are to analyze subtypes separately, or to examine whether patterns of mutual exclusivity are associated to these subytpes. We annotate results by known subtypes as a post-processing step in Multi-Dendrix.

### Somatic mutation data

We applied Multi-Dendrix and Iter-Dendrix to four somatic mutation matrices: (1) copy number variants (CNVs), small indels, and non-synonymous single nucleotide variants (SNVs) measured in 601 genes in 84 glioblastoma multiformae (GBM) patients [7]; (2) indels and non-synonymous single nucleotide variants in 623 sequenced genes in 188 Lung Adenocarcinoma patients [27]; (3) CNVs, small indels, and non-synonomous SNVs measured using whole-exome sequencing and copy number arrays in 261 GBM patients [7]; and (4) CNVs, small indels, and non-synonymous SNVs measured in 507 BRCA patients. We will refer to these datasets as GBM(2008), Lung, GBM, and BRCA below. We removed extremely low frequency mutations and known outliers from these datasets as described in Methods. After this processing, the GBM(2008) dataset contained mutation and CNV data for 46 genes in 84 patients; the Lung dataset contained somatic mutation for 190 genes in 163 patients; the GBM dataset contained mutation and CNV data for 398 genes in 261 patients; and the BRCA dataset contained mutation and CNV data for 375 genes in 507 patients. We focus here on presenting results from the latter two datasets because they are the latest whole-genome/exome datasets and most representative of the datasets that are now being produced and will be analyzed now and in the coming years. Results with the first two older and smaller datasets from targeted sequencing are described in the Supporting Information.

We compute 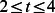 gene sets, each of minimum size 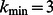 and maximum size ranging from 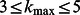. We summarize the results over these 9 different parameter values into modules using the procedure described above.

### Mutually exclusive sets in Glioblastoma (GBM)

We applied Multi-Dendrix and Iter-Dendrix to the GBM dataset, considering EGFR amplification as a separate event (see Methods). The algorithms report the same results over all values of the parameters except 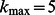, where Iter-Dendrix includes the IRF5 gene in a gene set with RB1, CDK4(A), and CDKN2A/CDKN2B(D), and MSL3. However, Multi-Dendrix is significantly faster running in 142 seconds compared to 37,786 seconds (over 10 hours) for Iter-Dendrix.

**Figure 2:**
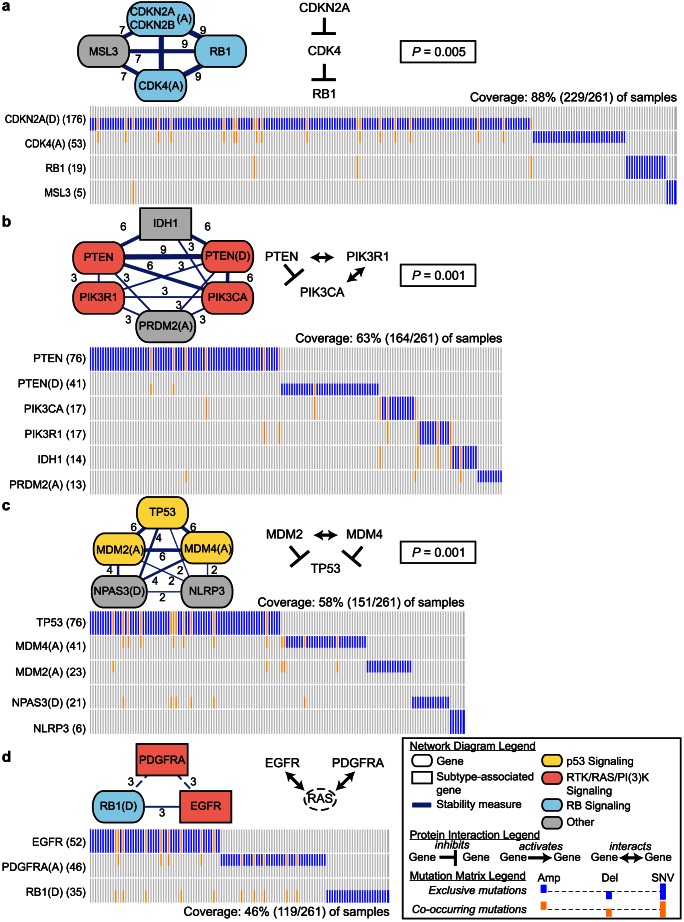
Multi-Dendrix results on the GBM dataset. (Left) Nodes represent genes in four modules found by Multi-Dendrix using 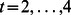 gene sets of minimize size 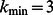 and maximum size 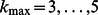. Genes with “(A)” appended are amplification events, genes with “(D)” appended are deletion events, and genes with no annotation are SNVs. Edges connect genes that appear in the same gene set for more than one value of the parameters, with labels indicating the fraction of parameter values for which the pair of genes appear in the same gene set. Color of nodes indicates membership in three signaling pathways noted in [7] as important for GBM: RB, p53, and RTK/RAS/PI(3)K signaling. Shape of nodes indicates genes whose mutations are associated with specific GBM subtypes, and dashed edges connect genes associated with different subtypes. The direct interactions statistic 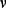 of this collection of gene sets is significant (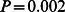). (Middle) Known interactions between proteins in each set and 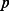-value for observed number of interactions. (Right) Mutation matrix for each of four modules with mutual exclusive (blue) and co-occurring mutations (orange).

We summarize the results of these different parameter choices by connecting genes that appear in the same gene set at least twice, resulting in four modules (Figure 2). These four modules include all the genes (except ERBB2) that are: (1) members of the three signaling pathways highlighted in the TCGA GBM study [7], and (2) are mutated in 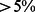 of the samples. The weight 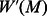 of all collections found by Multi-Dendrix on the GBM dataset are significant 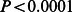) and the direct interactions statistic 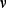 of these four modules is also significant (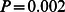). Three of the four modules also contain a significant number of interactions (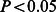). In addition to these four modules, two additional mutation classes, CNTNAP2 and deletion of 10q26.3, each appear in one choice of parameters for Multi-Dendrix. Since these are not part of a larger module, they are not analyzed further. Figure S2 shows a combined mutation matrix with all four modules.

The first module includes the amplification of CDK4, mutation of RB1, and a deletion that includes both CDKN2A and CDKN2B. This module is mutated in 87.7% (229/261) of the samples, and are discovered for all parameter choices. These four genes are members of the RB signaling pathway (as annotated in [7]) involved in G1/S progression (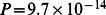 by Bonferonni-corrected hypergeometric test): CDKN2A and CDKN2B inhibits CDK4, which in turn inhibits RB1. In addition for 7/9 parameter choices, this module includes mutations in MSL3. MSL3 is a member of the MSL (male-specific lethal) complex that has a major role in dosage compensation in *Drosophila*. While this complex is conserved in mammals, the specific function of human MSL3 is unknown. However, the MSL complex also includes the histone acetyltransferase MOF which is involved in cell cycle regulation of p53 and may play a role in cancer [30]. Thus, the mutual exclusivity of mutations in MSL3 and the other well-known members of the RB signaling pathway is intriguing and deserves further study. This module contains two interactions (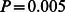).

The second module includes mutations and deletion of PTEN, mutations in PIK3CA, mutations in PIK3R1, mutations in IDH1, and an amplification that includes PDPN and PRDM2. The module is mutated in 62.8% (164/261) of the samples. PTEN, PIK3CA, and PIK3R1 are all members of the RTK/RAS/PI(3)K signaling pathway (as annotated in [7]) involved in cellular proliferation (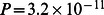). IDH1 is not a known member of this pathway; moreover, IDH1 is preferentially mutated in the proneural subtype of GBM [29]. Deletions in PTEN are also associated with the proneural subtype of GBM, although they are not considered a defining feature of this subtype (as IDH1 mutations are) and do not result in a gene expression signature [29]. However, there are no reports that PTEN, PIK3CA, or PIK3R1 mutations are subtype specific, and thus the mutual exclusivity of IDH1 and the remaining genes in this set is not simply explained by subtypes. PRDM2 is not known to be part of the RTK/RAS/PI(3)K signaling pathway. PRDM2 is a member of the histone methyltransferase superfamily, interacts with the RB protein [31], and is proposed as a tumor suppressor in colorectal cancer [32]. PDPN is used as a molecular marker for glioma, due to its association with clinical outcomes [33]. Our results suggest that PDPN and PRDM2 may have an undiscovered role in GBM as well. This module contains three interactions (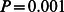).

The third module includes mutations in TP53, the amplification of MDM2, the amplification of MDM4, mutations in NLRP3, and the deletion involving AKAP6 and NPAS3. This module is mutated in 57.8% (151/261) of the samples, and appears for every parameter choice for 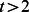. TP53, MDM2, and MDM4 are members of the p53 signaling pathway (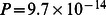), a critical and frequently altered pathway in GBM involved in senescence and apoptosis. NPAS3 is a transcription factor expressed in the brain and implicated in psychiatric disorders including schizophrenia [34], [35]. In addition, NPAS3 was recently shown to act as a tumor suppressor in astrocytomas, with a possible role in glioblastoma progression and proliferation [36]. This module contains three interactions (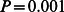).

The fourth module includes mutations in EGFR, the amplification of PDGFRA , and the deletion of RB1. This module is mutated in 45.6% (119/261) of samples, and appears for 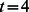. EGFR and PDGFRA are members of the RTK/RAS/PI(3)K signaling pathway (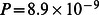), and RB1 is a member of the RB signaling pathway. While EGFR and PDGFRA both interact with RAS, there are no reported direct interactions between these three proteins. In addition, mutations in these three genes are significantly associated with two of the expression subtypes reported in [29]: mutations in EGFR and the deletion of RB1 are associated with the Classical GBM subtype, and the amplification of PDGFRA is significantly associated with the Proneural subtype. Thus, it appears that the mutual exclusivity discovered by Multi-Dendrix is a result of subtype-specific mutations, despite PDGFRA and EGFR being a member of the same biological pathway.

In summary, we see that subtype-specific mutations provide an alternative explanation for observed mutual exclusivity and confound the identification of driver pathways. However, on the GBM data subtype-specific mutations are a minor feature in the data, and Multi-Dendrix successfully identifies *de novo* portions of three critical signaling pathways in GBM.

### Mutually exclusive sets in Breast Cancer (BRCA)

We applied Multi-Dendrix and Iter-Dendrix to the BRCA dataset. We found that for most values of the parameters 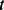 and 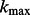, the results combined the most frequently mutated genes into a single gene set despite the fact that these genes had high coverage overlap (Figures S4 and S5). That is, for a gene set 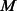, high coverage 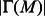 was outweighing a high coverage overlap 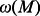 in the weight function 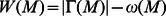 optimized by Multi-Dendrix. To enforce greater mutual exclusivity, we increased the coverage overlap penalty to 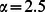 from its default value of 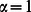.

**Figure 3:**
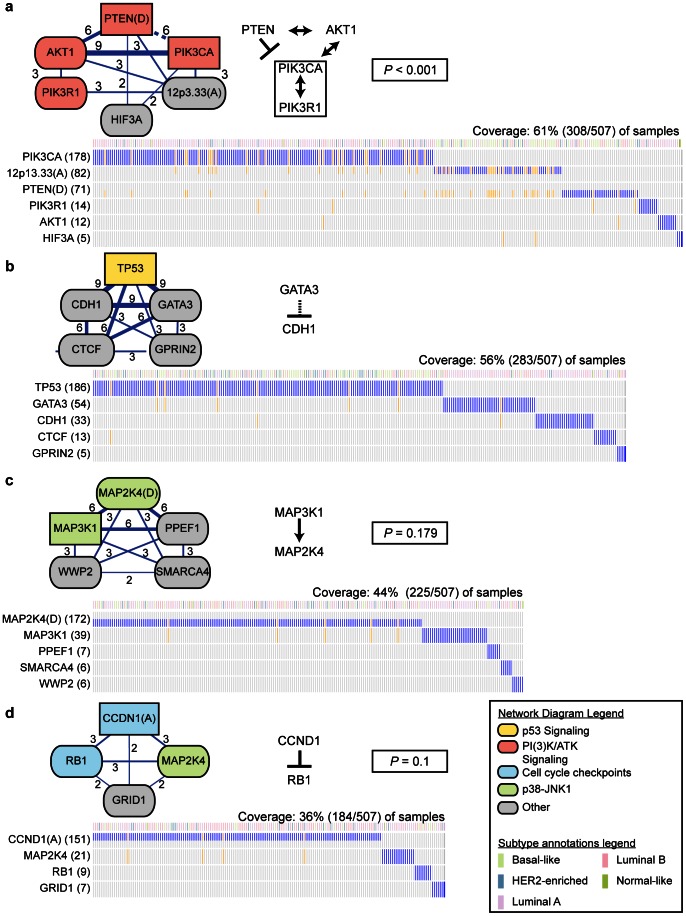
Multi-Dendrix results on the BRCA dataset. Graphical elements are as described in Figure 2 caption, except for the following. Color of nodes indicates membership in four signaling pathways noted in [8] as important for BRCA: p53 signaling, PI(3)K/AKT signaling, cell cycle checkpoints, and p38-JNK1. The top row of each mutation matrix annotates the subtype of each patient. The regulatory interaction between GATA3 and CDH1 is shown as a dashed line. The direct interactions statistic 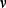 of this collection of gene sets is significant (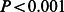).

Using 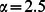, Multi-Dendrix identifies four distinct modules (Figure 3). These four modules overlap with three pathways known to be important in BRCA: p53 signaling, PI(3)K/AKT signaling, and cell cycle checkpoints. In addition, they include genes recently identified by [8] as important BRCA genes. These modules cover a smaller proportion of samples than our results on the GBM dataset even though they include the same number of genes, suggesting greater mutational heterogeneity or disease heterogeneity (i.e. subtypes) in the breast cancer dataset. Indeed, breast cancers are commonly divided into four major subtypes: Luminal A/B, Basal, and HER2 type. We annotate the subtype-specific mutations below, and Table S6 lists the significant associations between mutations and subtypes. The weight 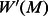 of all collections found by Multi-Dendrix on the BRCA dataset are significant (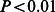), and the direct interactions statistic 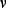 of these four modules is also significant (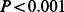). In addition, one module is significantly enriched for interactions (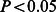). In addition to these four modules, five additional genes (SF3B1, CCDC150, COL23A1, C20orf26, PCDHA5) each appear in one choice of parameters for Multi-Dendrix. Since these are not part of a larger module, they were not further analyzed. Figure S3 shows a combined mutation matrix with all four modules.

The first module includes the deletion of PTEN, mutations in PIK3CA, PIK3R1, AKT1, HIF3A, and the amplification of genomic region 12p13.33. These six mutation classes are mutated in 61% (308/507) of the samples, and this module is discovered for all values of the parameters. PTEN, PIK3CA, PIK3R1, and AKT1 form the core of the PI(3)K/AKT signaling pathway, as annotated in [8] (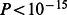): AKT1 is known to interact with PTEN, PIK3R1, and PIK3CA, while PTEN inhibits PIK3CA and PIK3R1. These genes constitute five of the eight genes mutated in the PI(3)K/AKT signaling pathway, as reported by [8]. One of the other mutation classes in this module is the amplification of 12p in 82 samples. This amplification event has been reported before in BRCA, although the likely target of this amplification is unknown [37]. This module contains six interactions (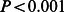).

The second module includes mutations in the genes TP53, CDH1, GATA3, CTCF, and GPRIN2. These five genes are mutated in 56% (283/507) of the samples, and are discovered for each parameter choice. TP53 is a member of the p53 signaling pathway, while GATA3, CDH1, and CTCF are well-known for their role in breast cancer and are involved in metastasis and proliferation. The loss or downregulation of CDH1, the E-cadherin gene located on 16q22.1, is implicated in breast cancer invasion and proliferation (reviewed in [38]). CTCF, also located on 16q22.1, has been found to act as a tumor suppressor in breast cancer through mechanisms similar to CDH1 [39]. GATA3 is a transcription factor that regulates immune cells, and has long been known to be involved in breast cancer tumorigenesis [40]. Recently, a novel role for GATA3 was discovered, whereby GATA3 suppresses breast cancer metastasis through inhibition of E-cadherin promoters [41]. This module contains zero known interactions.

The third module includes the deletion of MAP2K4 and mutations in MAP3K1, PPEF1, SMARCA4, and WWP2. These five mutations occur in 44.4% (225/507) of the samples, and this module is identified when the number 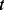 of gene sets is at least 3. MAP3K1 and MAP2K4 are both members of the p38-JNK1 stress kinase pathway as reported in [8], and are involved in the regulation of apoptosis. Both MAP3K1 and MAP2K4 are serine/threonine kinases, while PPEF1 is a serine/threonine phosphatase. The targets of PPEF1 are unknown, although there are reports that PPEF2, another member of the gene family, does interact with the p38-JNK pathway through the gene ASK1 [42]. SMARCA4 is also a known cancer gene, and has been shown to have a role as a tumor suppressor in lung cancer [43]. This module contains one interaction (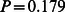).

The fourth module includes the amplification of CCND1 and mutations in MAP2K4, RB1, and GRID1. These four mutations occur in 36.3% (184/507) of the samples, and this module is discovered when the number 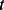 of gene sets is 4. CCND1 and RB1 encode interacting proteins that play a role cell cycle progression. CCND1 encodes the cyclin-D1 protein that inhibits the retinoblastoma protein encoded by RB1 via hyperphosphorylation. The hyperphosphorylation of *RB* inactivates its role as a tumor suppressor, and thus mutations that target either CCND1 or RB1 are thus important for proliferation in cancer [44]. Mutations in MAP2K4, as discussed above for the third module, target the p38-JNK1 pathway, and MAP2K4 is not known to have any interactions with CCND1 or RB1. This module contains one interaction (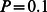).

We separately ran Multi-Dendrix on mutation data restricted to the 224 Luminal A patients, the 124 Luminal B patients, the 93 Basal-like patients, and the 58 HER2-enriched patients (as annotated in [8]) using different values of 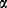 for the different sized datasets (see Supporting Information 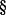 BRCA subtypes for details). The gene pair {TP53, GATA3} from the Multi-Dendrix modules is also identified by Multi-Dendrix when restricting to basal-like or luminal B samples. The same is true for the gene pair {AKT1, PIK3CA} in HER2-enriched and luminal A subtypes. Thus, the mutual exclusivity between mutations in these pairs of genes is not a result of subtype-specific mutations. On the other hand, the mutual exclusivity between 12p13.33 amplification and PIK3CA mutation appears to be an effect of subtypes, as these aberrations are associated with basal-like and luminal A subtypes, respectively (Table S6), and these two aberrations are not grouped into the same module on any Multi-Dendrix run of individual subtypes. These results show that mutual exclusivity can result from mutational heterogeneity within pathways, disease heterogeneity with subtype-specific mutations, or both.

## Discussion

We introduce an algorithm Multi-Dendrix that simultaneously finds multiple cancer driver pathways using somatic mutation data from a collection of patients. Multi-Dendrix finds combinations of somatic mutations solely from the combinatorial pattern of their mutations with *no prior knowledge of pathways or interactions between genes*. On simulated data, Multi-Dendrix outperforms iterative versions of Dendrix and RME, two previous algorithms for identifying single driver pathways. Multi-Dendrix finds optimal groups of mutually exclusive mutations even in the presence of significant noise where the passenger mutation rate is 15 times greater than observed in real data. Multi-Dendrix is orders of magnitude faster than iterative versions of Dendrix and RME and scales to the analysis of mutations in thousands of genes from hundreds of cancer patients. Finally, Multi-Dendrix finds optimal solutions over a range of sizes for the individual gene sets, while Dendrix examines only a fixed size gene set for each run.

We apply Multi-Dendrix to multiple cancer datasets, including glioblastoma and breast cancer data from The Cancer Genome Atlas, two of the most comprehensive somatic mutation datasets from high-throughput sequencing data. Multi-Dendrix finds multiple driver pathways of interacting genes/proteins including portions of the p53, RTK/RAS/PI(3)K, PI(3)K/AKT, and Rb signaling pathways. At the same time, we identify additional genes whose mutations are mutually exclusive with mutations in these pathways. Intriguingly, these additional genes are transcription factors or other nuclear proteins involved in regulation of transcription ( SMARCA4, NPAS3, PRDM2, and MSL3). In general, gene transcription is the downstream “output” of signaling pathways, suggesting that mutations in these downstream targets may be substituting for mutations in the upstream (and presumably more general) signaling pathways.

Our results demonstrate the advantages of simultaneously finding sets of mutually exclusive genes. However, there can be multiple explanations for observed mutual exclusivity in a set of genes including both pathways (i.e., interactions between genes) as well as disease heterogeneity (i.e., cancer subtypes). Interpretation of Multi-Dendrix results should consider these various explanations. To facilitate these analyses, we include additional steps in the Multi-Dendrix pipeline to compare the results sets of mutations to known gene interactions and/or known subtypes in the samples.

Our Multi-Dendrix analysis shows an interesting feature of mutual exclusivity between SNVs and deletions in tumor suppressor genes. In the glioblastoma data, we find that SNVs in PTEN and the deletion of PTEN are mutually exclusive. It is not clear whether this is a genuine biological feature of the mutational process, where either SNVs or deletions (but not both) are present in different samples, or merely an artifact of the mutation calling. Regarding the latter, it is possible that SNVs are more challenging to detect in hemizygous samples, where the other allele has been deleted.

There are several extensions that might further improve the Multi-Dendrix algorithm. First, the ILP used in Multi-Dendrix finds optimal solutions effectively, but is not guaranteed to rigorously examine suboptimal solutions. This is in contrast to the Markov Chain Monte Carlo (MCMC) approach used by Dendrix that samples suboptimal solutions in proportion to their weight. Extending the MCMC approach to simultaneous discovery of multiple pathways, or perhaps using the ILP to initialize a sampling procedure are interesting directions for future research. Second, the weight function used by the Multi-Dendrix algorithm does not explicitly incorporate co-occurrence of mutations between genes in different gene sets. Instead, the weight function is highest for gene sets with high coverage and approximate exclusivity, which may co-occur in many patients simply due to high coverage (a trivial example is when all gene sets have full coverage, and thus all mutations co-occur across gene sets). As noted in [25], co-occurrence of mutations is known to be important in large biological pathways, and thus algorithms that explicitly optimize for collections of gene sets where mutations between gene sets co-occur a surprising amount may have an advantage in identifying key components of these larger biological pathways.

We anticipate that Multi-Dendrix will be useful for analyzing somatic mutation data from different types of cancer and with larger cohorts of patients, such as those now being generated by The Cancer Genome Atlas (TCGA) and other large-scale cancer sequencing projects. However, mutual exclusivity and high coverage are not the only criteria for selecting driver mutations, and thus the model optimized by Multi-Dendrix has some limitations. As noted above, it is well-known that each patient has multiple driver mutations and there are many examples of co-occurring mutations. Some of these co-occurring mutations are clearly in different pathways, although this depends on one's definition of a pathway. Large, multi-functional “pathways” such as the cell-cycle pathway do indeed exhibit co-occurring mutations; on the other hand, co-occurring mutations appear to be less common in directly interacting genes/proteins. In addition, the coverage, or frequency, of important driver mutations may vary considerably, according to the stage at which patients are sequenced, or extensive disease heterogeneity. Private mutations that are unique to a single individual in a study might be driver mutations, but will not exhibit a strong signal of mutual exclusivity. Multiple approaches are required to prioritize somatic mutations for further experimental study. Multi-Dendrix is a useful complement for analysis of significantly mutated genes [6], [45], [46], functional impact [1]–[4], pathway/network analysis [18]–[22], and other approaches.

## Methods

### Weight function for gene sets

Given a mutation matrix 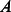, and a set 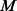 of genes (or equivalent mutation classes), Vandin et al. [23] define the weight function 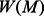 as follows. For a gene 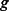, the *coverage* 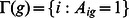 is the set of patients in which gene 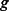 is mutated. Similarly, for a set 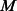 of genes, the *coverage* is 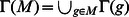. 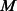 is *mutually exclusive* if 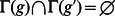, for all 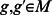, 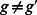. A gene set in 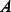 is a column submatrix of 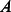 with high coverage and approximate exclusivity. Since increasing coverage may be achieved at the expense of decreasing exclusivity, [23] define a weight 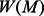 on set 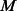 of columns of 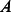 that quantifies both coverage and exclusivity of 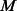. In particular, they define the *coverage overlap* of 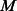 as 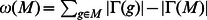. Note that 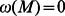 when 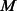 is mutually exclusive. Then 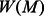 is the difference between the coverage and coverage overlap of 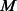:
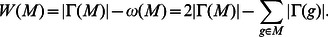
(2)Note that for a mutually exclusive submatrix 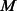, 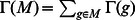 and therefore 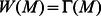.

Vandin et al. [23] introduce the Maximum Weight Submatrix Problem defined for an integer 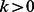 as the problem of finding the 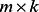 submatrix 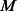 of 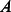 that maximizes a weight 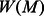, show that this problem is NP-hard and derive the De novo Driver Exclusivity (Dendrix) algorithm to solve it. Dendrix is a Markov Chain Monte Carlo (MCMC) algorithm that samples sets of 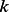 genes in proportion to their weight 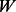. While the MCMC algorithm is not guaranteed to find a gene set of optimal 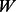, the authors showed that applying the method to real somatic mutation data produces gene sets with high coverage and approximate exclusivity, and that the Markov chain rapidly converges to a stationary distribution.

### An ILP for the Maximum Weight Submatrix Problem

We formulate the *Maximum Weight Submatrix Problem* as an integer linear program, which we refer to as 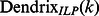. Given a mutation matrix 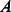 and gene set size 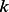, 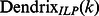 finds a gene set 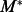 with largest weight 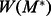. A gene set 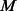 is determined by a set of indicator variables, one for each gene 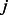,
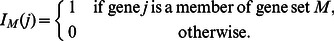
(3)To compute the weight function 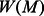 in (2), it is necessary to compute the coverage 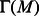. To do this, we define an indicator variable for each patient 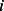,
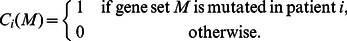
(4)Then, 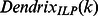 is defined as follows:
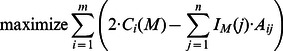
(5a)

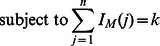
(5b)

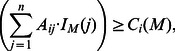
(5c)

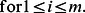
Note that the last constraint (5c) only forces 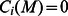 when all genes in 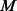 are not mutated (i.e. 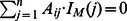), but does not force 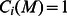 when at least one gene in 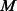 is mutated as required by (4). However, in the latter case the objective function will be maximized when 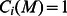 and thus (4) is satisfied.

Note that removing equation (5b) from 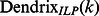 above produces a gene set with optimal 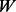 for any value of 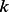, i.e. a gene set 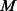 with maximum 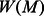 of any size. We will refer to this variant of the ILP as 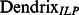. In addition, we can set bounds on the size of the gene set 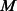, 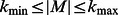, by replacing equation (5b) with
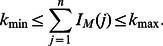
(6)We will refer to this variant as 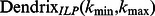.

### Multiple Maximum Weight Submatrices Problem

We define the following problem.

#### Multiple Maximum Weight Submatrices Problem

*Given an* 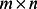 *mutation matrix* 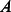 *and an integer* 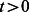 *, find a collection* 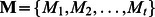 *of* 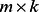 *column submatrices that maximizes* 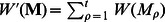.

Note that this problem is NP-hard, as stated above for the case 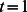. Further note that while the weight 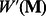 is increased by greater mutual exclusivity between mutations *within* a gene set, there is no restriction on the mutations *between* gene sets. Moreover, collections with large weight 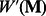 will also tend to have larger coverage 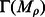 of each individual gene set 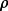. Thus, optimal solutions will tend to produce a collection with the property that many patients have a mutation in more than one gene set; or alternatively, there may be pairs or larger sets of co-occurring mutations, a phenomenon that has been observed in cancer [25].

We solve the *Multiple Maximum Weight Submatrix* problem using an integer linear program (ILP). We define an ILP, called Multi-Dendrix, using the same definitions of 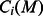 and 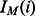 as above:
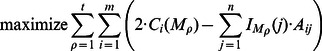
(7a)

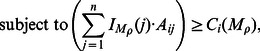
(7b)

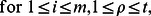


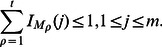
(7c)We solve this ILP using CPLEX v12.3 using default parameters. It is obvious that for a mutation matrix 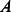, the collections 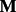 and 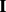 of gene sets produced by Multi-Dendrix and Iter-Dendrix, respectively satisfy 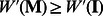. Multi-Dendrix can also produce sets 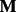 with strictly larger weight than the sets 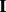 produced by Iter-Dendrix. There are a number of ways this might occur. First, there may be multiple gene sets 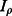 with maximum weight on iteration 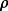, and only one of these is extended by the iterative algorithm. Second, the maximum weight gene set 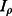 selected by Iter-Dendrix in the 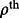 iteration may not be a member of the optimal 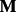; i.e. 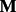 may contain gene sets that are suboptimal when considered in isolation. Third, when 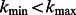 Multi-Dendrix may select gene sets with fewer than 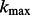 genes if this maximizes the weight 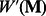 of the whole collection. We find that all of these cases occur on real somatic mutation data.

Multi-Dendrix can be extended to allow genes to be members of more than one gene set. Such genes may be involved in multiple biological processes. We define the parameter 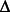 to be the maximum number of gene sets a gene can be a member of, and the parameter 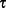 to be the number of overlaps allowed per gene set. Then we replace Eq. 7c with 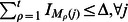 and add the constraint 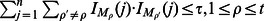. Note that the product in the second group of constraints must be encoded using additional indicator variables and thus the number of additional constraints grows rapidly as more overlaps between gene sets are allowed. While this extension is implemented in the Multi-Dendrix package, we did not use it for the results presented herein.

### Simulations

We ran Dendrix with default parameters using 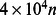 iterations for the MCMC method, where 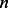 is the number of genes. We also used the default parameters for RME. We ran each algorithm with the true values 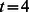 and 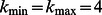. We compared the collection 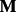 of gene sets found by each algorithm to the planted pathways 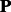, computing the difference between 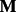 and 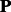 as follows:
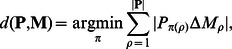
where 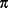 is a permutation of 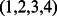 and 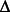 is the symmetric difference between sets.

### Construction of mutation matrices

We have two pipelines for building mutation matrices from somatic mutation data: one for the whole-exome datasets and the second for the targeted gene sequencing datasets, GBM(2008) and lung adenocarcinoma. Here we describe the pipeline for the whole-exome datasets. See Supporting Information for further details on the targeted gene sequencing data processing.

#### Whole-exome and copy number aberration data preparation

For the whole-exome datasets, we extracted non-silent somatic mutations from MAF files and copy number aberrations from GISTIC 2.0 [47] downloaded from the TCGA portal:

- GBM: http://gdac.broadinstitute.org/runs/analyses__2012_09_13/data/GBM/20120913/.
  MAF file: gdac.broadinstitute.org_GBM.Mutation_Assessor.Level_4.2012091300.0.0./GBM.maf.annotated.
  GISTIC wide peaks: gdac.broadinstitute.org_GBM.CopyNumber_Gistic2.Level_4.2012091300.0.0.tar.gz.
- BRCA: https://tcga-data.nci.nih.gov/docs/publications/brca_2012/.
  MAF file: “Somatic MAF archive [tar.gz] (public access)”.
  Segment data: “Merged segment file”.
  GISTIC wide peaks: “Genes in focal peaks [xslx]”.

We applied the following filters to remove genes from the analysis:

- 10,443 genes in the GBM dataset and 11,428 genes in the BRCA dataset mutated in fewer than 5 samples.
- 209 genes in the GBM dataset and 474 genes in the BRCA dataset whose coding regions are longer than 6 Kb and had fewer mutations than expected using a binomial test with an estimate of 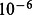 for the background mutation rate.
- 94 genes from both datasets that are observed to have unusually high rates of somatic mutation in exome-sequencing data, but are likely artifacts resulting from replication timing, active transcription, and other factors (as reported by M. Lawrence here: http://1.usa.gov/RBtuz7). These include olfactory receptors, “cub and sushi” proteins, and TTN.
- 10 additional genes (KRTAP5-5, HEATR7B2, EML2, CC2D1A, DUS3L, GABRA6, FLG, HYDIN, SUSD3, CROCCL1) that also appear to be artifacts as they were observed to have higher than average mutation rates, but with no known role in cancer.

For copy number data, we used the “wide peaks” in the GISTIC output as the copy number aberrations in each sample, with copy number ratio thresholds of 0.1 and −0.1 for determining if a segment is amplified or deleted, respectively. We performed the following additional filtering of the copy number data.

- We combine copy number aberrations that co-occur in the same sample 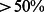 of the time and are within 10 Mb of one another into a single larger “meta” gene.
- We restricted analysis to 249 CNVs in the GBM data and 204 CNVs in the BRCA data that appeared in wide peaks with 20 or fewer genes or were given a peak label by GISTIC.
- For BRCA, we removed segments longer than 10 Mb.
- Remove wide peaks containing known fragile sites at PARK2 [48], WWOX [49], RPL5, and FAM19A5.

Note that in many cases, the wide peaks include multiple genes which we combine into a single “meta” gene for Multi-Dendrix analysis. We manually selected the target gene for the following metagenes:

- For GBM, we selected CDK4, CDKN2A and CDKN2B, PTEN, PDGFRA, MDM2, MDM4, PIK3R1, and RB1 as the targets of their respective aberrations.
- For BRCA, we used the GISTIC peak labels as the target for each aberration.
- In the GBM dataset, we treated EGFR as a separate event and did not include it in the Multi-Dendrix analysis. EGFR amplification is the most common amplification, and second most common mutation (after CDKN2A/B deletion). There is a significant co-occurrence between EGFR amplifications and SNVs (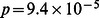 by Fisher's exact test), although EGFR amplification occurs in 74 more samples than EGFR SNV.

After this filtering, we analyze a total of 398 mutation classes in GBM and 375 mutation classes in BRCA (i.e. separating SNVs and CNVs in individual genes or metagenes).

### Evaluating known interactions

We assess whether the mutually exclusive gene sets found by Multi-Dendrix are enriched for interacting genes using the following *direct interactions test*. We use two analogous tests: one test for collections, and one test for for individual gene sets.

For a collection 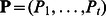, let 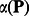 be the fraction of possible interactions between pairs of genes *within* each gene set 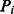 that are observed and 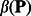 be the fraction of possible interactions between pairs of genes in *different* pairs of gene sets 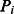, 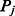 that are observed. We define 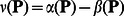. Thus, a large value 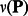 indicates a collection with many interactions between genes in the same gene set and few interactions between genes in different gene sets. This measure models our assumptions that mutual exclusivity should be strongest between genes that directly interact. Moreover, we also expect few interactions between genes in different gene sets reported by Multi-Dendrix. Thus, the measure 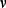 is a more strict measure of the topology of interactions than merely counting the total number of observed interactions. Similarly, we assess an individual gene set by counting the number of interactions among genes in that gene set. Let 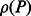 be the number of interactions among genes in 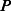.

We assess the statistical significance of 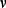 and 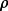 by comparing the observed value to the empirical distribution of 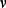 and 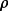 on an ensemble of permuted networks with the same degree distribution. We permute the network by swapping edges between pairs of genes, so as to preserve the distribution of edges within the network. We perform 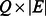 edge swaps, where 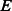 is the edges in the network and 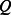 is some constant (we use 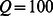 as recommended for permuting graphs with fixed degree distribution in [50]). This network permutation corrects for the observation that many genes that are frequency mutated in cancer also have many interactions in human PPI networks. Since frequently mutated genes typically appear in Multi-Dendrix results, the number of interactions between genes in Multi-Dendrix results might be higher than a *random set* of genes of the same size. When performing the direct interactions test, we use a protein-protein interaction (PPI) network constructed from the union of interactions reported in KEGG [13] and iRefIndex 9.0 [51] containing 236,060 interactions among 15,257 proteins. We calculate 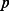-values from 1000 permuted versions of this combined network.

By examining only direct interactions between genes in our sets, we might miss cases where genes do not directly interact, but have a common interacting partner (e.g. EGFR, PDGFRA, and NF1 all share RAS as an interacting partner in the fourth module of the GBM results). Average pairwise distance is another commonly used metric for assessing whether groups of genes are clustered on an interaction network, and this metric might identify such cases. However, the tradeoff is that the diameter (average pairwise distance) of most biological interaction networks is small, and thus many genes are close on the network. We found that average pairwise distance was not a strict enough measure for examining mutually exclusive gene sets (data not shown). Finally, note that counting the number of interactions between genes in a PPI network is only an approximate measure of biological function. Current interaction networks have large number of false positive interactions – particularly when interactions from high-throughput experiments are included – as well as an unknown number of false negatives. In addition, there is a problem of ascertainment bias as cancer genes are some of the most studied human genes and many of their interactions have been assessed.

## Supporting Information

Figure S1Multi-Dendrix runtimes on simulated datasets. Average runtime (secs) of Multi-Dendrix on simulated data with 1000 patients and varying passenger mutation probabilities 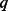 and number of genes. For each set of parameters, average runtime across 10 simulations is reported. The average runtime of Multi-Dendrix is under 3500 seconds for each dataset.(EPS)

Figure S2 Multi-Dendrix results on GBM dataset as one mutation matrix. The four modules are shown in alternating gray backgrounds. For each module, patients are sorted first by those with mutations in only the module (dark blue), then by patients that have just one mutation in the module (light blue), and finally by patients that have co-occurring mutations in that module (orange). Upticks indicate amplification, downticks indicate deletions, and full ticks indicate SNVs. The four modules found by Multi-Dendrix on the GBM dataset cover 98.8% (258/261) of the patients in the GBM mutation data.
(EPS)

Figure S3 Multi-Dendrix results on BRCA dataset as one mutation matrix. Representation is as in Figure 5. The four modules found by Multi-Dendrix cover 91.9% (466/507) of the patients in the BRCA mutation data.
(EPS)

Figure S4Multi-Dendrix results on the BRCA dataset with 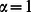. Four genes (genes with degree zero) are output for only one choice of parameter values. The remaining genes form two connected components indicating that the collections are variable across different values of the parameters. In comparison Multi-Dendrix with 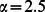 (Figure 3) finds more stable results on this dataset.(EPS)

Figure S5Iter-Dendrix results on the BRCA dataset. The modules identified by Iter-Dendrix group the most frequently mutated genes together (e.g. TP53 and PIK3CA) and thus there is a large number of samples with co-occurring mutations in each module. In comparison Multi-Dendrix with 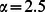 (Figure 3) finds more stable with less co-occurrence of mutations within modules on this dataset.(EPS)

Figure S6 Multi-Dendrix results on the GBM(2008) dataset.
(EPS)

Figure S7 Multi-Dendrix results on the Lung dataset.
(EPS)

Figure S8 Multi-Dendrix results on the BRCA dataset restricted to Basal-like patients.
(EPS)

Figure S9 Multi-Dendrix results on the BRCA dataset restricted to Luminal A patients.
(EPS)

Figure S10 Multi-Dendrix results on the BRCA dataset restricted to Luminal B patients.
(EPS)

Figure S11 Multi-Dendrix results on the BRCA dataset restricted to HER2-enriched patients.
(EPS)

Figure S12 Multi-Dendrix results on the unfiltered GBM mutation data.
(EPS)

Figure S13 Multi-Dendrix results on the unfiltered BRCA mutation data.
(EPS)

Table S1Average number of genes input to Multi-Dendrix across 100 simulated datasets. We use a minimum threshold of 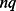, the expected number of 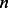 total samples with a mutation. We report the values for different number 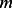 of genes and the lowest and (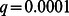) highest (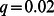) passenger mutation probabilities. On all simulated datasets, Multi-Dendrix 's average runtime is 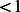 hour.(PDF)

Table S2

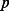
-values for the number of observed protein-protein interactions in Multi-Dendrix results (direct interactions test) for different values of parameters 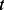, the number of gene sets, and 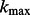, the maximum gene set size. The minimum gene set size, 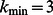 for all runs. The 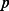-values were calculated from 1000 permuted networks constructed from the union of the KEGG and iRefIndex PPI networks.(PDF)

Table S3

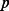
-values for the number of observed protein-protein interactions in Iter-Dendrix results (direction interactions test) for different values of parameters 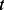, the number of gene set, and 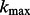, the maximum gene set size. The minimum gene set size, 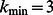 for all runs. The 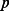-values were calculated from 1000 permuted networks constructed from the union of the KEGG and iRefIndex PPI networks.(PDF)

Table S4Significant associations (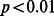) between mutations in genes (SNVs, amplifications “(A)”, or deletions “(D)”) and three subtypes from GBM consensus clusters. 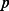-values were calculated using Fisher's exact test with a Bonferroni correction for multiple hypotheses.(PDF)

Table S5Significant associations (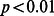) between mutations (SNVs, amplifications “(A)”, or deletions “(D)”) in the four BRCA subtypes. 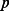-values were calculated using Fisher's exact test with a Bonferroni correction for multiple hypotheses.(PDF)

Table S6Gene sets found by Iter-RME in the GBM(2008), GBM, and BRCA datasets (after removing genes with mutation frequency 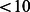
%) for maximum gene set size 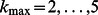 and and number 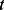 of gene sets 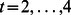. For all values of 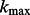, Iter-RME returned only gene sets of size 2. “Iteration” column denotes the index of each gene set 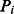 returned in each iteration of RME. Only 4/12 gene sets contain an interacting pair of genes according to the union of the KEGG and iRefIndex protein-protein interaction network.(PDF)

Table S7 Gene sets found by Iter-RME in the GBM(2008), GBM, and BRCA datasets (after removing genes with mutation frequency <10%) for maximum gene set size kmax = 2,…,5 and and number t of gene sets t = 2,…,4. For all values of kmax, Iter-RME returned only gene sets of size 2. “Iteration” column denotes the index of each gene set Pi returned in each iteration of RME. Only 4/12 gene sets contain an interacting pair of genes according to the union of the KEGG and iRefIndex protein-protein interaction network.
(PDF)

Text S1 Further analyses of the mutation data and comparisons of Multi-Dendrix, Iter-Dendrix, and RME.
(PDF)
