# Supplementary material for: Simultaneous Identification of Multiple Driver Pathways in Cancer
Source: PLoS Comput Biol. 2013 May 23;9(5):e1003054. doi: 10.1371/journal.pcbi.1003054 (PMC3662702; doi:10.1371/journal.pcbi.1003054)
Supplement: Table S1 — Average number of genes input to Multi-Dendrix across 100 simulated datasets. We use a minimum threshold of , the expected number of total samples with a mutation. We report the values for different number of genes and the lowest and () highest () passenger mutation probabilities. On all simulated datasets, Multi-Dendrix 's average runtime is hour. (PDF) [file pcbi.1003054.s014.pdf]

m	**100**	**200**	**400**	**800**	**1600**	**3200**	**6400**	**12800**	**22000**
$q = 0.02$	51.8	93.4	185.4	357.8	722.7	1409.3	2826.6	5663.4	9698.4
$q = 0.0001$	24.2	34.9	54	88.1	169.1	312.9	617.2	1236	2108.4
