# Supplementary material for: Simultaneous Identification of Multiple Driver Pathways in Cancer
Source: PLoS Comput Biol. 2013 May 23;9(5):e1003054. doi: 10.1371/journal.pcbi.1003054 (PMC3662702; doi:10.1371/journal.pcbi.1003054)
Supplement: Table S2 — -values for the number of observed protein-protein interactions in Multi-Dendrix results (direct interactions test) for different values of parameters , the number of gene sets, and , the maximum gene set size. The minimum gene set size, for all runs. The -values were calculated from 1000 permuted networks constructed from the union of the KEGG and iRefIndex PPI networks. (PDF) [file pcbi.1003054.s015.pdf]

	GBM(2008)			GBM			BRCA		
$k_{\max}$	3	4	5	3	4	5	3	4	5
$t = 2$	0.005	0.026	0.027	< 0.001	< 0.001	< 0.001	0.003	0.007	0.006
$t = 3$	< 0.001	0.021	0.375	< 0.001	0.001	0.001	< 0.001	0.003	0.007
$t = 4$	0.253	0.036	0.314	0.013	0.006	0.002	< 0.001	0.001	0.01
