# Supplementary material for: Simultaneous Identification of Multiple Driver Pathways in Cancer
Source: PLoS Comput Biol. 2013 May 23;9(5):e1003054. doi: 10.1371/journal.pcbi.1003054 (PMC3662702; doi:10.1371/journal.pcbi.1003054)
Supplement: Table S4 — Significant associations () between mutations in genes (SNVs, amplifications “(A)”, or deletions “(D)”) and three subtypes from GBM consensus clusters. -values were calculated using Fisher's exact test with a Bonferroni correction for multiple hypotheses. (PDF) [file pcbi.1003054.s017.pdf]

	**GBM(2008)**			**GBM**			**BRCA**		
$k_{\max}$	3	4	5	3	4	5	3	4	5
$t = 2$	0	0	3	0	0	1	6	8	10
$t = 3$	6	6	13	0	0	1	9	12	15
$t = 4$	4	10	15	0	0	1	12	16	20
