# Supplementary material for: Simultaneous Identification of Multiple Driver Pathways in Cancer
Source: PLoS Comput Biol. 2013 May 23;9(5):e1003054. doi: 10.1371/journal.pcbi.1003054 (PMC3662702; doi:10.1371/journal.pcbi.1003054)
Supplement: Table S5 — Significant associations () between mutations (SNVs, amplifications “(A)”, or deletions “(D)”) in the four BRCA subtypes. -values were calculated using Fisher's exact test with a Bonferroni correction for multiple hypotheses. (PDF) [file pcbi.1003054.s018.pdf]

Gene	Subtype	p-value
IDH1	Consensus 3	1.24E-05
ARID2(D)	Consensus 3	3.66E-05
PDGFRA(A)	Consensus 3	3.79E-05
CDK4(A)	Consensus 3	0.00098192
KIF4B	Consensus 3	0.001198772
BBS1	Consensus 3	0.006889494
TP53	Consensus 3	0.008230181
MET(A)	Consensus 3	0.009878469
