# Supplementary material for: Simultaneous Identification of Multiple Driver Pathways in Cancer
Source: PLoS Comput Biol. 2013 May 23;9(5):e1003054. doi: 10.1371/journal.pcbi.1003054 (PMC3662702; doi:10.1371/journal.pcbi.1003054)
Supplement: Table S6 — Gene sets found by Iter-RME in the GBM(2008), GBM, and BRCA datasets (after removing genes with mutation frequency %) for maximum gene set size and and number of gene sets . For all values of , Iter-RME returned only gene sets of size 2. “Iteration” column denotes the index of each gene set returned in each iteration of RME. Only 4/12 gene sets contain an interacting pair of genes according to the union of the KEGG and iRefIndex protein-protein interaction network. (PDF) [file pcbi.1003054.s019.pdf]

Gene	Subtype	p-value
TP53	Basal-like	1.53E-18
12p13.33(A)	Basal-like	2.17E-10
PTEN(D)	Basal-like	3.16E-06
MAP3K1(D)	Basal-like	7.93E-06
5q21.3(D)	Basal-like	0.000109176
PIK3CA(A)	Basal-like	0.000417525
RB1(D)	Basal-like	0.001671689
11p13(A)	Basal-like	0.002899894
FGF2(A)	Basal-like	0.003068744
EPS8L1	Basal-like	0.00649918
MYC(A)	Basal-like	0.007955101
IGF1R(A)	Basal-like	0.008512299
ERBB2(A)	HER2-enriched	5.07E-26
TP53	HER2-enriched	8.64E-09
MIR21(A)	HER2-enriched	4.24E-08
6q21(A)	HER2-enriched	0.001672642
4q13.3(A)	HER2-enriched	0.002513158
SRPR	HER2-enriched	0.003786332
ATP1A4	HER2-enriched	0.007586379
ERBB3	HER2-enriched	0.007586379
PIK3CA	Luminal A	7.39E-06
MAP3K1	Luminal A	9.94E-05
CCND1(A)	Luminal B	1.36E-08
KCNB2	Luminal B	0.001123083
3p25.1(A)	Luminal B	0.001333521
