# Supplementary material for: Simultaneous Identification of Multiple Driver Pathways in Cancer
Source: PLoS Comput Biol. 2013 May 23;9(5):e1003054. doi: 10.1371/journal.pcbi.1003054 (PMC3662702; doi:10.1371/journal.pcbi.1003054)
Supplement: Table S7 — Gene sets found by Iter-RME in the GBM(2008), GBM, and BRCA datasets (after removing genes with mutation frequency <10%) for maximum gene set size kmax = 2,…,5 and and number t of gene sets t = 2,…,4. For all values of kmax, Iter-RME returned only gene sets of size 2. “Iteration” column denotes the index of each gene set Pi returned in each iteration of RME. Only 4/12 gene sets contain an interacting pair of genes according to the union of the KEGG and iRefIndex protein-protein interaction network. (PDF) [file pcbi.1003054.s020.pdf]

Dataset	Iteration	Genes	Interact
GBM(2008)	1	RB1, CDKN2B	No
	2	NF1, EGFR	No
	3	OS9, CDKN2A	No
	4	TP53, MDM2	Yes
GBM	1	CDK4(A), CDKN2A_CDKN2B(D)	Yes
	2	PTEN, PTEN(D)	N/A
	3	MDM4(A), TP53	Yes
	4	EGFR, PDGFRA(A)	No
BRCA	1	TP53, GATA3	No
	2	PIK3CA, PTEN(D)	Yes
	3	MAP2K4(D), FOXA1(A)	No
	4	8p11.23(A), ERBB2(D)	No
