# Supplementary material for: Simultaneous Identification of Multiple Driver Pathways in Cancer
Source: PLoS Comput Biol. 2013 May 23;9(5):e1003054. doi: 10.1371/journal.pcbi.1003054 (PMC3662702; doi:10.1371/journal.pcbi.1003054)
Supplement: Text S1 — Further analyses of the mutation data and comparisons of Multi-Dendrix, Iter-Dendrix, and RME. (PDF) [file pcbi.1003054.s021.pdf]

Supporting Information

GBM(2008) and Lung datasets

Somatic mutation processing in targeted gene sequencing studies.

For the GBM(2008) and lung adenocarcinoma datasets, we used all genes with non-synonymous single-nucleotide mutations or small indels in at least two patients. For GBM(2008), we also included copy number variants (CNVs), adding a CNV mutation type for a gene if the gene had a CNV of the same type (amplification or deletion) in at least 90% of the patients with a CNV. If a set of genes was mutated in the same patients, we merged these genes into a single metagene. We manually merged CYP27B1 into the metagene containing CDK4, as described in [23].

Multi-Dendrix results

We ran Multi-Dendrix and Iter-Dendrix on the GBM(2008) and Lung datasets using the same parameters our analysis of the GBM and BRCA datasets: minimum gene set size $k_{\min} = 3$, maximum gene set size $k_{\max} = 3 \ldots 5$, number t of gene sets $t = 2 \ldots 4$, for a total of 9 parameter combinations. We analyze Multi-Dendrix's results below, and present a comparison of Multi-Dendrix's and Iter-Dendrix's results in § Comparison of Multi-Dendrix and Iter-Dendrix results.

GBM(2008). Figure S6 shows Multi-Dendrix's results on the GBM(2008) dataset. Multi-Dendrix finds four modules that contain known cancer genes. In addition eight genes appear in the results for one or two parameter choices, but with different groups of genes. Two modules contain triples that are consistent across all parameter choices: $\{$CDKN2B, RB1, CDK4$\}$, and $\{$CDKN2A, TP53, DTX3$\}$. Note that due to different copy number processing, the genes CDKN2A and CDKN2B appear as different mutation classes in the GBM(2008) data, while they were merged in a metagene in the GBM data presented above. Beyond this difference, the triple $\{$CDKN2B, CDK4, RB1$\}$ is the same as the triple in the first module found by Multi-Dendrix on the GBM dataset, and contains two known interactions (for further analysis of this module, see § Mutually exclusive sets in Glioblastoma (GBM)). For six of the nine parameter choices, this module also contains the well-known cancer gene ERBB2, a member of the RTK pathway important for glioblastoma [7].

The second triple, $\{$CDKN2A, TP53, DTX3 $\}$, contains two frequently mutated GBM cancer genes that interact: TP53 and CDKN2A. For six of the nine parameter choices, this module also contains CDC123, although CDC123 is mutated in only two samples. The third module contains both EGFR and NF1, two genes identified by [29] to be associated with the Classical and Mesenchymal subtypes, respectively. These two genes account for 52 of the 70 mutated samples in this module, and thus the exclusivity of mutations in this module is likely due to subtype-specific mutations. Interestingly, the two genes associated with the Proneural subtype, IDH1 and PDGFRA, are not found in any of the results (IDH1 was not included in the list of targeted genes for the GBM(2008) dataset). The weight W' of all collections identified by Multi-Dendrix are significant ($p < 0.0001$).

Lung. Figure S7 shows Multi-Dendrix's results on the Lung dataset. In contrast to the other datasets, the output of Multi-Dendrix varies widely with the choice of parameters (e.g. STK11 has 8 edges with weight ≥ 0.2 in the summarization graph). There are two large modules: one of size four and one of size thirteen, as well as 10 additional genes that appear with another gene for only one choice of parameters (and thus are nodes of degree 1 in the summarization graph).

The module of size four includes the triple TP53, ATM, and PAK4 found for all values of parameters. This triple is nearly perfectly exclusive; it covers 79 patients (i.e. $\Gamma(M) = 79$) with a coverage overlap $\omega(M) = 1$. TP53 and ATM are both tumor suppressors that interact in response to DNA damage, and

have been shown previously to be recurrently mutated in lung adenocarcinoma [52]. PAK4 is involved in both cellular survival and angiogenesis, and has been implicated in tumor progression in a variety of cancers [53], though we did not find reports of its role in lung adenocarcinoma. While PAK4 is mutated in only three patients in the Lung dataset, it is still significantly exclusive with ATM ($p = 0.0008$ by Fisher's exact test), and may deserve further inquiry.

The module of size 13 includes two gene triples that are grouped together for most parameter choices. The triple $\{$EGFR, KRAS, EPHB1$\}$ contains the well-known interaction between EGFR and KRAS, two proteins whose mutations are important for lung adenocarcinoma [27]. Similar to EGFR, EPHB1 is a receptor tyrosine kinase, and mutations in this gene (as in mutations in EGFR) were reported to be associated with higher copy number and mRNA expression levels in [27]. The triple $\{$STK11, NF1, NTRK1$\}$ contains two well-known cancer genes: STK11 and NF1. Mutations in STK11 have long been a hallmark of lung adenocarcinoma, but the discovery of mutations in the tumor suppressor NF1 was more recent [27]. While the genes in this triple have no known interactions, the triple is nearly perfectly exclusive (coverage $\Gamma(M) = 54$; coverage overlap $\omega(M) = 1$) which indicates that lung adenocarcinoma patients need inactivating mutations in just one of these genes. Thus, they may have an uncharacterized relationship in lung adenocarcinoma.

While Multi-Dendrix's results on the Lung dataset are promising, we did not conduct further analysis or comparison of these results because of the inconsistency across parameter choices. We note that a likely explanation for this inconsistency is that the processed data includes a relatively small number of 163 samples and only includes mutations from targeted sequencing of 190 genes, missing other mutations and copy number aberrations in these samples.

Comparison of Multi-Dendrix and Iter-Dendrix results

We compare the gene sets found by Multi-Dendrix and those found by Iter-Dendrix on the GBM(2008), GBM, and BRCA datasets, computing the overlap between the results of the algorithms and comparing the number of known protein-protein interactions in the results.

Overlap

We compared the distances between collections found by Multi-Dendrix and Iter-Dendrix, using the symmetric difference function $d(\mathbf{M}, \mathbf{I})$ defined in § Simulated data. Table S4 reports these distances. There are many differences between the two algorithms on the GBM(2008) and BRCA datasets. On the GBM dataset, there is only one difference: Iter-Dendrix includes the gene IRF5 in the gene set that includes RB1, CDK4(A), and CDKN2A/CDKN2B(D) for $k_{\max} = 5$.

Interactions

Tables S2 and S3 show the results of the direct interactions test on collections found by Multi-Dendrix and Iter-Dendrix (for details of the test, see § Evaluating known interactions). In total, across all three datasets, nearly all collections found by Multi-Dendrix are significantly enriched for interactions (24/27 collections, $p < 0.05$), slightly more than are found by Iter-Dendrix (20/27 collections, $p < 0.05$). The largest difference is on the BRCA dataset, where all collections found by Multi-Dendrix have lower p-values than Iter-Dendrix. In addition, Multi-Dendrix finds all nine collections significantly enriched for interactions ($p \leq 0.05$), while Iter-Dendrix finds just two. This difference is amplified by the fact Iter-Dendrix finds five collections with $p \geq 0.15$ (by comparison the largest p-value for Multi-Dendrix on BRCA is 0.01). Thus, overall Multi-Dendrix finds collections of gene sets that are more enriched for interactions.

We do not compare the enrichment for interactions of individual gene sets found by Multi-Dendrix and Iter-Dendrix. Iter-Dendrix is a greedy algorithm and is thus guaranteed to find the same gene sets multiple

times as we vary t. Thus, it is more reasonable to evaluate the stable modules for enrichment for interactions, as presented in the § Mutually exclusive sets in Glioblastoma (GBM) and § Mutually exclusive sets in Breast Cancer (BRCA).

Comparison of Multi-Dendrix and RME

We compared Multi-Dendrix and Iter-RME on the GBM(2008), GBM, and BRCA datasets. We ran RME with default parameter values, including the default minimum mutation frequency of $\geq 10\%$. After removing genes mutated in fewer than 10% of samples, the GBM(2008), GBM, and BRCA datasets contains 18, 10, and 28 genes, respectively. As a result, using the same parameters as above, RME can find at most three gene sets in the GBM dataset of the minimum size 3, since there are only 10 genes with mutation frequencies $\geq 10\%$ the GBM dataset. We ran RME for up to $t = 4$ gene sets of size $k_{\max} \in [2, 5]$ so that RME could find $t = 4$ gene sets in the GBM and BRCA data. Note that unlike Dendrix, RME has a parameter for setting the maximum gene set size, equivalent to $k_{\max}$, and returns gene sets of size $[2, k_{max}]$. Thus, at each iteration, we chose the gene set $P, 2 \leq |P| \leq k_{\max}$, such that P had the largest RME algorithmic significance score.

For all values of the parameters t and $k_{\max}$, Iter-RME finds collections where each gene set has size 2. This is due to a combination of RME's algorithmic significance score, which is highest for gene sets of size 2, and also because there are only 10-28 genes in each dataset. It is difficult to compare Multi-Dendrix and Iter-RME using the tests presented in Section , as Iter-RME finds only 4 gene sets of size two in the GBM(2008), GBM, and BRCA datasets. It is unclear how interesting these pairs are: only three pairs of genes from all datasets interact in either the iRefIndex or KEGG protein-protein interaction networks, despite these pairs being chosen from the most highly mutated genes in each dataset (Table S7). Thus, the runtime requirements of RME limit its use to only a small subset of of highly mutated genes, and precludes RME from finding interesting gene sets of size greater than 2 on the tested datasets. In contrast, with the same parameters, Multi-Dendrix finds larger gene sets of up to 5 genes, many of which show enrichment for interactions (see Results).

Subtype analysis

GBM subtypes

We annotate the Multi-Dendrix results for associations with known subtypes. [29] derived four subtypes of GBM from gene expression data, and these classifications have become the standard in TCGA analysis. However, we were unable to directly use the sample annotations from [29] for the GBM mutation dataset since there are only 39 samples in common between the two datasets. Instead, we identify whether the gene sets produced by Multi-Dendrix contain any of the subtype-specific genes (IDH1, PDGFRA, EGFR, and NF1) reported in [29]. Figure 2 shows that one of the four modules found by Multi-Dendrix contains three of these four genes (all except IDH1). These genes are all members of the RTK/RAS/PI(3)K signaling pathway as annotated in [7], although they do not interact in iREFIndex or KEGG.

We also considered associations between subtypes defined by other automated clustering of gene expression data from TCGA. We downloaded GBM subtypes generated from consensus hierarchical clusters of mRNA expression data from the Broad's Firehose website. This dataset contains subtype information for 529 samples, 224 of which appear in our GBM mutation dataset, and maps patients to one of three subtypes ("1", "2", or "3"). Note that [29] identified four subtypes, and thus there is no one-to-one correspondence between the two subtype classifications. We determined if mutations in a gene were associated with subtype using Fisher's exact test. Table S5, lists all significant associations ($P < 0.01$ following Bonferroni multiple hypothesis correction). Notably, Consensus 1 and Consensus 2 have no significant associations, while the Consensus 3 subtype has all eight of the the significant associations. These include the markers IDH1

and PDGFRA in the Proneural subtype of [29]. The other two subtype markers found by [29], EGFR and NF1, which characterize the Classic and Mesenchymal subtype, do not appear associated with any of the Consensus clusters. Because the overlap between these clusters and those of [29] was imperfect, we used the genes that [29] reported to have subtype-specific mutations.

BRCA subtypes

We ran Multi-Dendrix on BRCA mutation data restricted to patients from each of the subtypes detailed in [8]. Because the number of patients within each subtype is not distributed evenly, we varied Multi-Dendrix's exclusivity parameter α for each subtype (Luminal A: $\alpha = 2$; Luminal B: $\alpha = 1.5$; HER2-enriched: $\alpha = 1$; Basal-like: $\alpha = 1$). We discuss these results in § Mutually exclusive sets in Breast Cancer (BRCA). In addition, we report all significant associations (significance level: $p < 0.01$) between genes or mutation classes and BRCA subtypes in Table S6.

Multi-Dendrix results without SNV filtering

We tested Multi-Dendrix on the GBM and BRCA mutation matrices constructed without any filtering of SNV date. Thus, we included all 10,987 (respectively 12,248) genes with at least one non-synonymous SNV in any sample. We also included the copy number aberrations output from GISTIC as described in § $Construction of mutation matrices$. The resulting mutation matrices included 11,023 mutation classes in 261 GBM samples and 12,281 mutation classes in 507 BRCA samples. We report the stable modules identified by Multi-Dendrix here. The modules identified on the mutation data without SNV processing have symmetric distance Δ of 7 and 36 from the modules identified from the mutation data with SNV processing. In the GBM data, the differences are minor: for all four modules reported in the main text, at least four genes are in common with the corresponding module found with the unfiltered mutation data. In particular, the Multi-Dendrix results with the unfiltered data contain the following changes:

- LMNB2 and SUSD3 appear in the module containing CDKN2A(D), CDK4(A), RB1, and MSL3.
- KRTAP5-5 replaces NLRP3 in the module containing TP53, MDM4(A), MDM2(A), and NPAS3(D).
- HMCN1 replaces PIK3R1 for two parameter choices in the module containing PIK3CA, PTEN(D), PTEN, IDH1, and PRDM2(A).
- MDN1 appears in the module containing EGFR, PDGFRA(A), and RB1(D).

On the BRCA data, the differences are larger: for three of the four modules reported in the main text, only two of the genes in those modules are placed in the same module when Multi-Dendrix is run on the unfiltered mutation data. However, the fourth module (TP53, GATA3, CDH1, CTCF, and GPRIN2) remains exactly the same. In particular, the Multi-Dendrix results with the unfiltered data contain the following changes:

- HUWEI, SVIL, and LPHN3 replace AKT1, PIK3R1, 12p13.33(A), and HIF3A in the module containing PTEN(D) and PIK3CA.
- LAMA2 and USP34 replace MAP2K4 and GRID1 in the module containing CCND1(A) and RB1.
- TLN1, MYH7, DNAH1, and NOTCH4 replace SMARCA4, PPEF1, and WWP2 in the module containing MAP2K4(D) and MAP3K1.

Many of the genes that appear only in the modules found with unfiltered data are long genes or genes with high background mutation rates that are more likely to be mutated in more samples than other genes.

The fifteen genes that only appear in modules on the unfiltered GBM and BRCA data have an average length of greater than 8500, and an average BMR of greater than 5×10^{-6} (BMRs were calculated from SNV data from twelve cancers). Such outlier genes are a potential source of false positives, not only for Multi-Dendrix, but also for any method that attempts to identify cancer genes by their recurrence across samples [6]. Multi-Dendrix identified the stable modules in 6,863 and 14,491 seconds, respectively.

References

52. Greulich H (2010) The genomics of lung adenocarcinoma: opportunities for targeted therapies. Genes & cancer 1: 1200-10.

53. Kesanakurti D, Chetty C, Rajasekhar Maddirela D, Gujrati M, Rao JS (2012) Functional cooperativity by direct interaction between PAK4 and MMP-2 in the regulation of anoikis resistance, migration and invasion in glioma. Cell death & disease 3: e445.
